# Participation of Actin on *Giardia lamblia* Growth and Encystation

**DOI:** 10.1371/journal.pone.0007156

**Published:** 2009-09-23

**Authors:** Araceli Castillo-Romero, Gloria Leon-Avila, Armando Perez Rangel, Rafael Cortes Zarate, Carlos Garcia Tovar, Jose Manuel Hernandez

**Affiliations:** 1 Departamento de Biologia Celular, CINVESTAV-IPN, Mexico Distrito Federal, Mexico; 2 Departamento de Parasitologia, Escuela Nacional de Ciencias Biologicas del Instituto Politecnico Nacional, Mexico Distrito Federal, Mexico; 3 Departamento de Microbiologia y Patologia, Universidad de Guadalajara, Guadalajara Jalisco, Mexico; 4 Departamento de Ciencias Biologicas, UNAM, FES-Cuautitlan, Cuautitlan Izcalli, Mexico; Charité-Universitätsmedizin Berlin, Germany

## Abstract

**Background:**

Microfilaments play a determinant role in different cell processes such as: motility, cell division, phagocytosis and intracellular transport; however, these structures are poorly understood in the parasite *Giardia lamblia*.

**Methodology and Principal Findings:**

By confocal microscopy using TRITC-phalloidin, we found structured actin distributed in the entire trophozoite, the label stand out at the ventral disc, median body, flagella and around the nuclei. During *Giardia* encystation, a sequence of morphological changes concurrent to modifications on the distribution of structured actin and in the expression of actin mRNA were observed. To elucidate whether actin participates actively on growth and encystation, cells were treated with Cytochalasin D, Latrunculin A and Jasplakinolide and analyzed by confocal and scanning electron microscopy. All drugs caused a growth reduction (27 to 45%) and changes on the distribution of actin. Besides, 60 to 80% of trophozoites treated with the drugs, exhibited damage at the caudal region, alterations in the flagella and wrinkles-like on the plasma membrane. The drugs also altered the cyst-yield and the morphology, scanning electron microscopy revealed diminished cytokinesis, cysts with damages in the wall and alterations in the size and on the intermembranal space. Furthermore, the drugs caused a significant reduction of the intensity of flourescence-labeled CWP1 on ESV and on cyst wall, this was coincident with a reduction of CWP1 gene expression (34%).

**Conclusions and Significance:**

All our results, indicated an important role of actin in the morphology, growth and encystation and indirectly suggested an actin role in gene expression.

## Introduction

*Giardia lamblia* causes giardiasis worldwide, the infection may course asymptomatic but frequently it causes severe diarrhea, the principal symptom of the disease. During its life cycle, *Giardia lamblia* presents two stages: the trophozoite and the cyst. The trophozoites are responsible for the symptomatology [1], [2], [3]. The cyst is the infective form, it is very resistant to the environment and can be viable for two or three months in humid places at 4 to 8°C [4]. The trophozoite becomes a cyst as a consequence of some stimuli such as environmental pH changes and/or the concentration of bile salts or cholesterol in the intestine. Morphological and biochemical changes occur as a consequence of still poorly understood signaling which include the synthesis of specific new antigens and the biogenesis of new secretory compartments called encystation specific vesicles (ESV). ESV transport the cyst wall proteins (CWP) and precursor components to the surface of the newly formed cysts [5], [6], [7], [8], [9], [10], [11].

*Giardia lamblia* cytoskeleton is particular [12]. It is considered neither as complex as the eukaryote cytoskeleton nor as simple as the bacteria [13]. By treating this parasite with neutral detergents an insoluble cytoskeleton fraction (Triton cytoskeleton) is obtained, composed by around 20 proteins from 20 to 200 kDa. The main components are tubulin and giardins, constituing 40–50% of *Giardia*'s cytoskeleton proteins [14], [15], [16], [17]. Other *Giardia* cytoskeleton proteins, like actin, have been reported by using heterologous antibodies [18], [19].

Microfilaments are very dynamic structures, some actin perturbing drugs have been used as crucial tools to elucidate the microfilament functions [20], [21]. In higher eukaryotes, they are determinant for muscular contraction, shape and cell growth, intracellular transport, phagocytosis, preservation of the flatened shape of Golgi cisterna, endocytosis, and mitosis [22], [23], [24], [25]. In *Giardia lamblia*, actin has been found on the periphery of the ventral disk, the median body and between the nuclei, where the basal bodies and axonems are located [18], [19]. Previous reports have shown that cytochalasins interfere with *Giardia* adhesion *in vitro* [26], [27], [28], inhibit *Giardia* growth and cause morphological alterations [29]. The mentioned data suggest that the microfilaments participate in *Giardia's* adhesion and growth. In this study we demonstrated, by using microfilament disturbing drugs, that actin plays a critical role in growth and encystation, it is important in morphology and indirectly regulates CWP1 gene expression. Our results could aim to establish new strategies focused to find specific targets to avoid the formation of cysts, the infective form of *Giardia*.

## Methods

### Culture of *Giardia lamblia* and *in vitro* encystation

Axenic cultures of *Giardia lamblia* (WB strain) trophozoites were grown at 37°C in Diamond's TYI-S-33 medium pH 7.1 supplemented with 10% bovine serum and 0.5 mg/ml bovine bile [30]. For *in vitro* encystation, 6×10^5^ trophozoites/ml were cultured at 37°C for 24 h in TYI-S-33 medium pH 7.8, supplemented with 10% bovine serum and 10 mg/ml bovine bile. Trophozoites and cysts were diluted in phosphate-buffered saline (PBS) and counted in a hemocytometer.

### Analysis of actin distribution on trophozoites and during in vitro encystation

*Giardia lamblia* trophozoites (6×10^5^ cells/ml) were cultured at 37°C for 24 h in TYI-S-33 encystation medium. The distribution of actin was analyzed at the begining of encystation (time 0, trophozoites) and during the encystation process at 6, 12, 18 and 24 h by using TRITC-phalloidin. The stained samples were analyzed by confocal microscopy.

### *In vitro* experiments with drugs

In order to evaluate the effect of microfilament disturbing drugs on *Giardia lamblia* growth, 10^3^ trophozoites were grown for 24 h, then, cells were incubated with 10 µM Cytochalasin D (CD; Sigma-Aldrich St. Louis, Mo. USA) or 1 µM Latrunculin A (LA, ; Sigma-Aldrich St. Louis, Mo. USA) or 1 µM Jasplakinolide (JAS; Calbiochem-Novabiochem La Jolla, CA. USA) disolved in 0.1% DMSO. Cultures were monitored for 24 to 72 h. 0.1% DMSO, the drugs diluent was used as a negative control. To prove the effect of the same drugs on the encystation process, trophozoites (6×10^5^) were cultured for 24 h in encystation medium containing 10 µM CD, 1 µM LA, or 1 µM JAS. The doses used for each compound were selected accordingly to previous studies in *Giardia*, some other parasites and mammalian cells. [20], [21], [26], [29], [31], [32].

### Expression and purification of CWP1

The cyst wall protein 1 (CWP1, GenBank Accession No. U09330 ) gene was amplified from genomic DNA by PCR using the primers: forward 5′-AGA AGA GAA TTC AAA TGA TGC TCG CTC TCC TTG C-3′ and reverse 5′-TCT TCT GCG GCC GCT TTC AAG GCG GGG TGA GGC AG-3′ (*EcoR*I and *Not*I restriction sites are underlined). The product was cloned into pPROEX-1 expression vector in order to get pPROEX-1-CWP1 and after that DH5α *E. coli* was transformed with this construction. The construction was verified by DNA sequencing (Automated sequencer ABI Prism 310, Perkin-Elmer, Applied Biosystems) and the overexpression of the fusion protein was induced by adding 1 mM isopropyl-D-thiogalactopyranoside IPTG (Invitrogen Carlsbad, CA. USA) to the transformed cells culture. Fusion protein was purified by Ni-NTA Agarose affinity chomatography (Qiagen; Valencia, CA USA), following the manufacturer instructions. Protein concentration was determined by Bradford assay [33] and purity was analyzed by 12% SDS-PAGE [34].

### Production of policlonal antibodies

BALB/c mice and Wistar rats were immunized with 100 µg and 500 µg of recombinant CWP1, respectively, by intraperitoneal route at 0, 7 and 15 days. First immunizations were applied as an emulsified mixture 10∶1 protein∶TiterMax® (Sigma) [35]; the following challenges were done with a mixture 1∶1 protein∶aluminum hydroxide. Pre-immune and immune sera were assayed by ELISA and Western blot [36], [37]. All the procedures involving animals were carried on following federal and local regulations for animal care and use (CINVESTAV-IACUC, approved by the Mexican Oficial Norm: NOM-062-ZOO-1999).

### Immunofluorescence

Cells were washed twice with PBS and fixed for 1 h at 37°C with 1% paraformaldehyde in PBS. Fixed cells were washed twice with PBS, applied on coverslips precoated with poly-L-lysine and were allowed to adhere at room temperature. Then, cells were permeabilized with 0.5% Triton X100-SDS for 30 min, washed three times with PBS and incubated at room temperature for 30 min with 1% bovine serum albumin. Then, cells were labeled with diluted 1∶100 TRITC-phalloidin (Sigma) for 1 h. Cysts were processed as trophozoites, but they were incubated for 1 h with primary polyclonal anti-CWP antibody (1∶300) before phalloidin labeling. Then, the cells were washed twice with PBS and incubated for 1 h with diluted 1∶100 TRITC-phalloidin and 1∶100 Cy5 conjugated anti-mouse (Jackson ImmunoResearch Laboratories). Finally, coverslips were mounted on glass slides with Vectashield mounting medium (Vector Laboratories) and analyzed by Confocal Microscopy (Leica DMIRE2 and TCS-SPE). The images were processed with the Leica Lite and the Leica Application Suite softwares and transformed to the apropriate quality format.

### Relative-quantitative RT-PCR

The actin (GenBank accession no. AAA99305.1 and CWP1 (GenBank accession no. U09330) cDNAs were synthesized, by a reverse transcriptase reaction (INVITROGEN), using 2 µg of RNA (purified by Trizol method) from trophozoites and cysts and the following primers: actin sense 5′-AGA AGA GAA TTC AAA TGA CAG ACG ACA ACC CTG CCA TAG-3′ and actin antisense 5′-TCT TCT GCG GCC GCT TCA CAT ACA CTT ACG GTT TGC AAT G-3′.and CWP1 sense 5′-TCT TCT GCG GCC GCT TCA CAT ACA CTT ACG GTT TGC AAT G-3′ and CWP1 antisense 5′-TCT TCT CCA TGG TAG GCG GGG TGA GGC AGT ACT CTC CGC AGT CCG-3′ Relative-quantitative RT-PCRs were performed in a 7500 Real Time PCR System (Applied Biosystems, Foster City CA. USA), using Maxima™ SYBR Green qPCR Master Mix (2X) (Fermentas Life Sciences) to monitor the amplification reactions. The expression of both genes were normalized to the expression of *Giardia* glyceraldehyde 3-phosphate dehydrogenase (*gap1*) gene (GenBank accession no. M88062) using gap1 sense primer 5′-GCA AGC GTG TCA TCA TCT CCG CTC CG-3′ and gap1 antisense primer 5′-AAG GAC CTT CCC GAC AGC CTT TGC G-3′. Also a melting curve was performed to confirm the absence of primer dimerization. The relative-quantitative RT-PCR conditions were: hot start 95°C for 10 min, 40 cycles at 95°C for 30 s, 65°C for 30 s and 72°C for 1 min. Expression data were determined by using the comparative ΔΔCt method. Significant differences (p<0.05) (calculated by *t*–Student and ANOVA tests using the program GraphPad Prism 5.02) are indicated by asterisks in figures. Error bars indicate standard deviation for experiments with more than one trial.

### Scanning electron microscopy

For scanning electron microscopy (SEM) analysis, trophozoites and cysts were washed and fixed with 2.5% glutaraldehyde in PBS for 1 h. Then, cells were adhered on poly-L-lysine pretreated coverlips, washed three times with PBS and post-fixed in 1% osmium tetroxide for 1 h. Next, cells were washed with PBS, dehydrated in alcohol, dried to critical-point with CO_2_, coated with gold and analyzed in a SEM (JSM-35C).

## Results

### Actin is distributed in the entire trophozoite, median body, disc, flagella and concentrated into threads at the central part of the cyst

Even though there are no available homologous antibodies against *Giardia* actin, some authors have revealed the localization of this protein in *Giardia* trophozoites by using heterologous antibodies [18], [19], but there is no a specific analysis that shows the distribution of actin during the encystation process.

**Figure 1:**
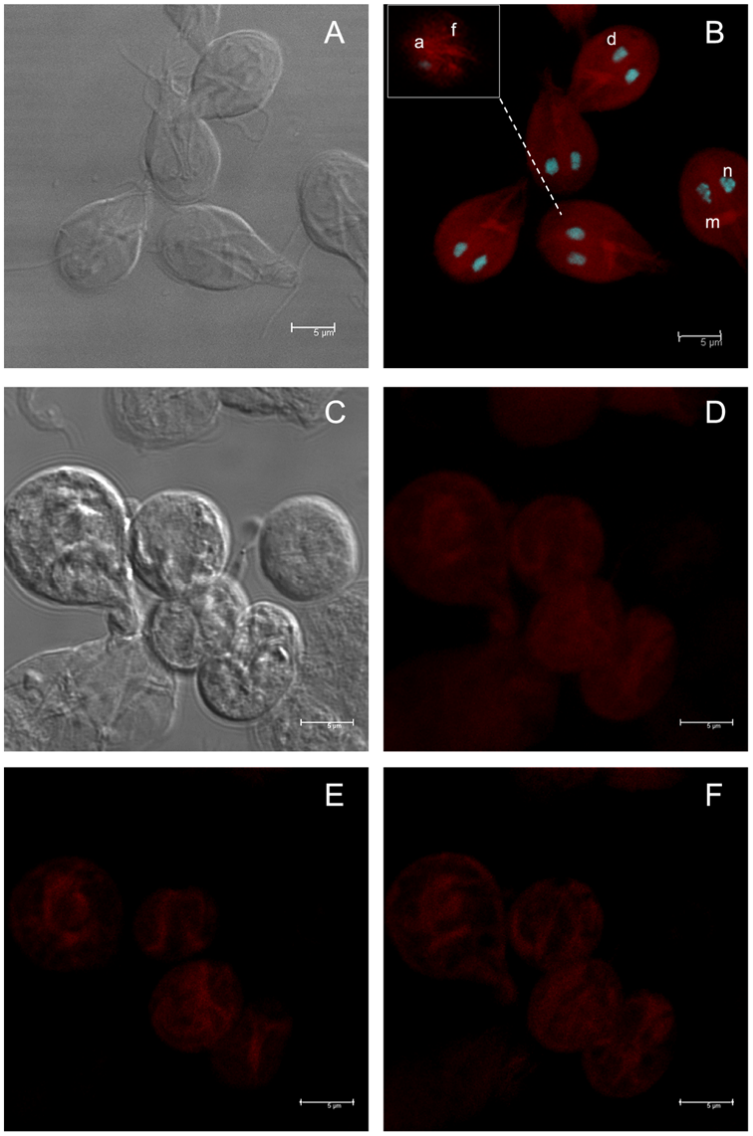
Localization of actin in *Giardia lamblia* trophozoites and cysts. The cells were labeled using TRITC-phalloidin and analyzed by confocal microscopy. A, DIC image of trophozoites. B, Trophozoites stained with TRITC-phalloidin (d = ventral disc; m = median body; n = nuclei; and, f = flagella). C, DIC image of cysts. D, Cysts stained with TRITC-phalloidin. E and F, optical slices of D. The B inset represents an optical slice. The nuclei were labelled with DAPI.

In order to detect actin in both stages of *Giardia*, we first analyzed its distribution using TRITC-phalloidin –a well accepted specific tool for structured actin identification–. Confocal microscopy images of trophozoites (Figure 1A) demonstrated actin distributed like small spots in the entire trophozoites and in nuclei. However an intense stain was evident, in median body, on the periphery of the ventral disc and flagella (Figure 1B). In cysts (Figure 1C), actin was distributed as thick ribbons forming a compact oval structure, also some patches were evidents (Figure 1D–F).

### Morphological changes in the encystation process involved remodeling of actin

**Figure 2:**
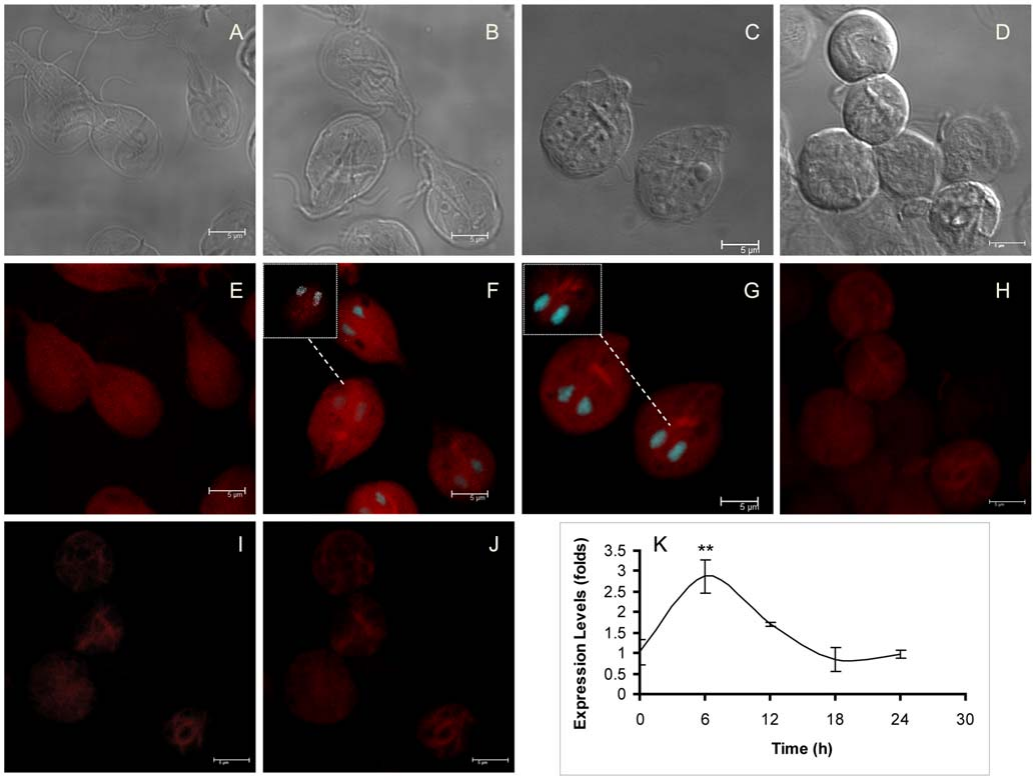
Distribution and expression of actin during encystation of *Giardia lamblia*. Parasites cultured in encystation medium were stained with TRITC-phalloidin along the encystation process at time points 0 h (E), 12 h (F), 18 h (G) and 24 h (H). I and J, are optical slices of H. A–D, are the corresponding DIC images for E–H respectively. The insets in F and G are optical slices of the marked cells. The nuclei were labelled with DAPI. In the F inset, the white points represent the colocalization beetween DAPI and phalloidin. K, relative-quantitative RT-PCR analysis of actin expression at the same encystation times. Values are presented as fold change over time zero, **p = 0.0026.

As early as 6 h after encystation process began, trophozoites became round and progressed to oval mature cysts transformation in the next 24 h. A sequence of morphological changes were observed by light microscopy during *Giardia* encystation. The most visible or evident changes occurred at 12, 18, and 24 h ( Figure 2B–D).

In order to evaluate the distribution of actin along the encystation process, actin was stained with TRITC-phalloidin at 0, 12, 18 and 24 h, and then analyzed by confocal microscopy. The most evident changes of actin distribution arose since the 12 h, at this time point, the ESV become visible; the labeled actin appeared more condensed, grouped in some patches, around the ESV and in nuclei (Figure 2B, F). As encystation proceeded to 18 h, the number of ESV increased and the staining in the median body increased in comparison to non-encysting cells (Figure 2A, E). The stained actin became smooth and uniformly scattered in the body of precysts, excluding the area occupied by the ESV. However a bright stain was evident in the proximal region of the ventral axonems. (Figure 2C, G). After 24 h, actin was irregularly distributed in the core of mature cysts (Figure 2H), it was found forming patches (Figure 2I, J). Besides, during *Giardia* encystation the relative-quantitative RT-PCR analysis demostrated variations of actin mRNA expression; at 6 h of encystation, actin mRNA increased almost two folds in comparison to non encysting cells, after, the expression decreased and remaining almost constant from 18 to 24 h of encystation (Figure 2K).

### Cytochalasin D, Latrunculin A and Jasplakinolide impair *Giardia lamblia* growth and encystation process

**Figure 3:**
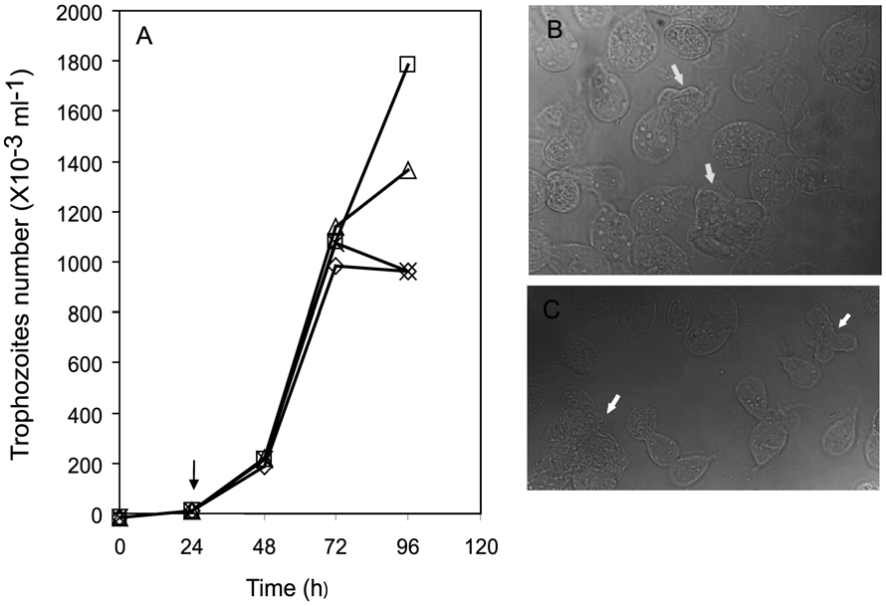
Effect of CD, LA and JAS on *Giardia lamblia* growth and encystation. A, trophozoites cultured in presence of 10 µM CD (X), 1 µM LA (◊) and 1 µM JAS (Δ). The arrow in the figure indicates the time when the compounds were added. DMSO (□), the diluent of the compounds was used as control. B and C, DIC images from parasites cultured for 24 h in encystation medium containing 10 µM CD. Note the damages caused by the drug, depicted with white arrows.

Some studies suggest the participation of actin in *Giardia* growth, in ceramide endocytosis and in its adherence to cell lines and glass [26], [27], [28], [29], [38]; however, there are no specific descriptions or studies focused on the role of actin in *Giardia* growth and encystation. In order to address these specific questions, drug-response and time-course curves were performed in *Giardia lamblia* cultures in the presence of actin disrupting drugs (CD, LA and JAS). All drugs affected the *Giardia* growth. The effect of drugs on growth curve was observed since the 24 h post-treatment and the maximum effect for CD and LA was achieved after 48 h (Figure 3A). At 72 h, a 27 to 45% yield reduction of the parasites was observed. Besides, the drugs were added at the beginning of the encystation process, the newly-formed precysts and cysts were also affected. There was an unusual sticky aggregation of the cysts that make difficult the quantification of these *Giardia* forms. Nevertheless, there were an increment in precyst forms and damaged cells (Figure 3B,C).

### Microfilament disorganizing drugs caused: actin redistribution, morphological changes on trophozoites and cysts and indirectly CWP1 down regulation

**Figure 4:**
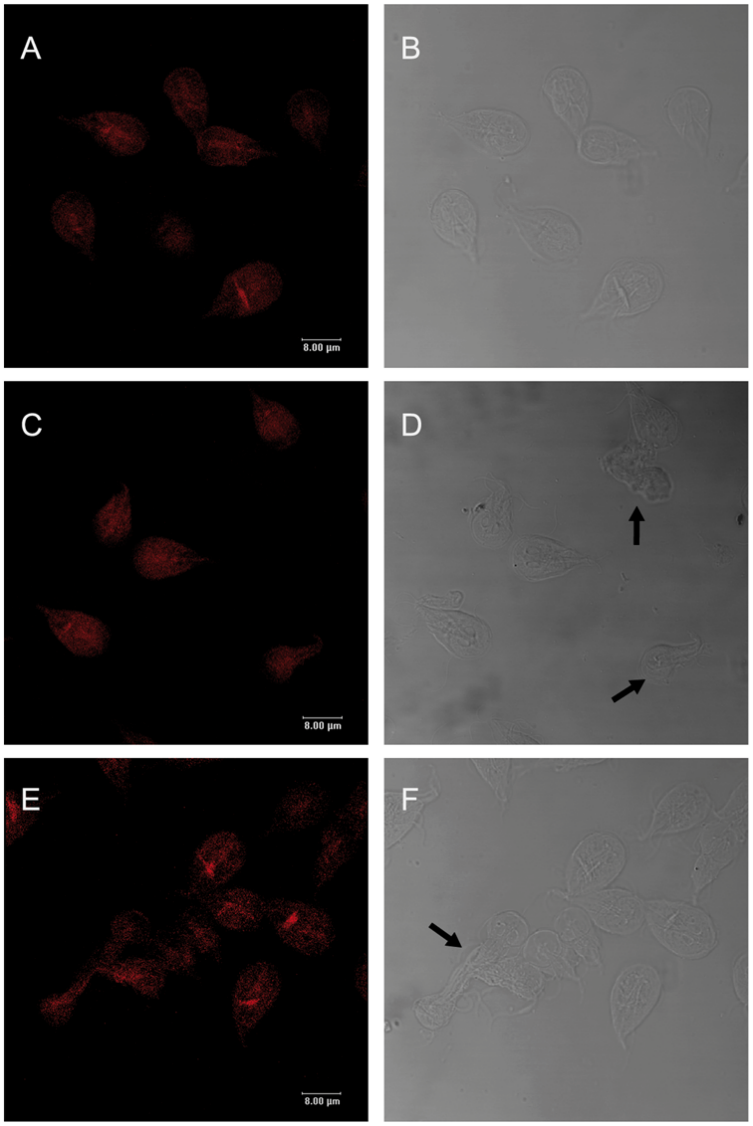
Effects of CD and LA on *Giardia* trophozoites. Cells were treated with 10 µM CD (C and D) and 1 µM LA (E and F) for 72 h and stained with TRITC-phalloidin, the DIC images are B,D and F. DMSO, the diluent of the drugs was used as a control (A and B). The black arrows point out some trophozoites with morphological alterations

Because CD and LA had the higher effect on growth and encystation, we continued the study with these drugs to visualise the actin cytoskeleton destabilization in *Giardia*. After drug treatment, the cells were stained with TRITC-phalloidin and analyzed by confocal mycroscopy. At 72 h of treatment, the drugs caused visible morphological changes on trophozoites; several cells became rounded, some others (30%) were retracted and the major proportion, about 80–90% of the cells, had the caudal region damaged (Figure 4D and F) in comparison to DMSO-treated cells (Figure 4A and B). Furthermore, the actin cytoskeleton destabilization also was manifested by a dispersed dimmed label of actin. We observed that those parasites with extreme damage lost the phalloidin staining (Figure 4C and E). Besides, LA caused a distribution of actin like light dots in the body and an intense label at the median body (Figure 4E).

**Figure 5:**
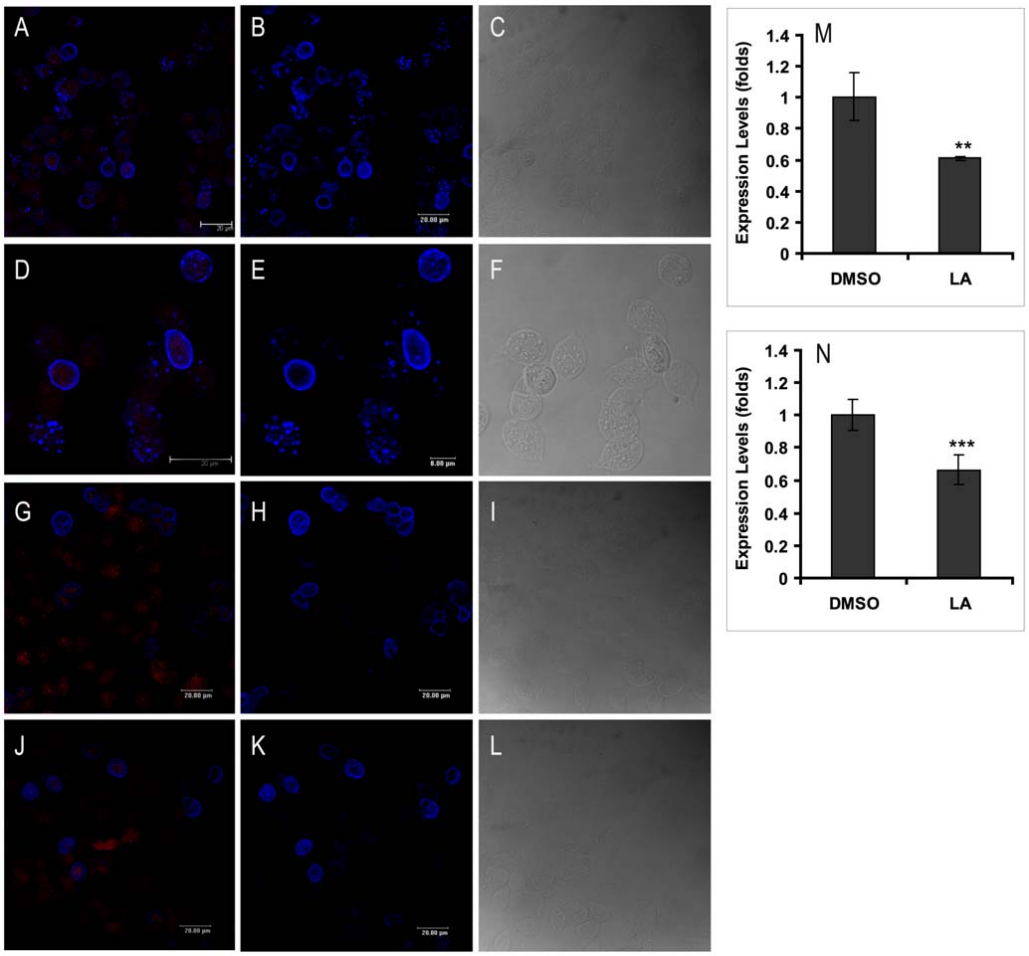
Effects of CD and LA on *Giardia* cysts. The cells were treated with 10 µM CD (G–I) and 1 µM LA (J–L) for 24 h and stained with TRITC-phalloidin (red) and anti-CWP1 (blue). DMSO was used as a control (A–F). The superposition of actin and CWP1 images are A, D, G and J. M and N, relative-quantitative PCR analysis of actin and CWP1 expression, respectively. Values are presented as fold change over time zero, **p = 0.0011, ***p<0.0001.

On the other hand, the encystation process of *Giardia lamblia* is complex; it involves biochemical and morphological changes [9], [10] in which undoubtedly the cytoskeleton participates but the molecular mechanisms involved remain in study. In order to analyze whether actin has a role in encystation, this process was induced in the presence of the same compounds and an anti-CWP1 was used as a marker to differentiate precysts from cysts. After the drug-treatment, several parasites exhibited morphological alterations at the membrane, in the disc and in the caudal region. Some cysts showed either an increased or decreased size and formed unusual aggregates (Figure 5I and L). The drugs also caused changes on actin staining patterns, it was also detected a reduction of parasites harboring ESV (Figure 5G, H, J and K). Besides, a relative-quantitative RT-PCR analysis demonstrated that LA provoked a 34% of down expression of CWP1 mRNA (Figure 5N), coincident to a reduction of 44% of actin mRNA expression (Figure 5M).

### Microfilament disturbing drug-induced morphological damage on *Giardia lamblia* trophozoites and cysts

**Figure 6:**
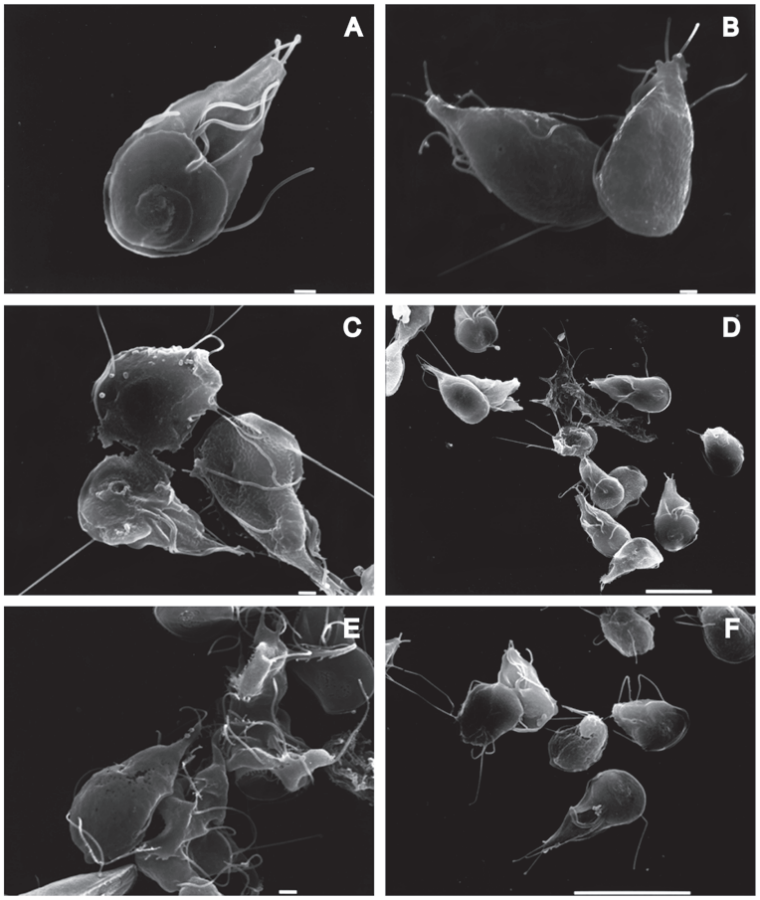
SEM micrographs of *Giardia lamblia* trophozoites treated with CD and LA. Samples of treated trophozoites, were processed for SEM as described in methods. 10 µM CD (C and D), 1 µM LA (E and F), DMSO control samples (A and B). Bars are 1 and 10 µm.

**Figure 7:**
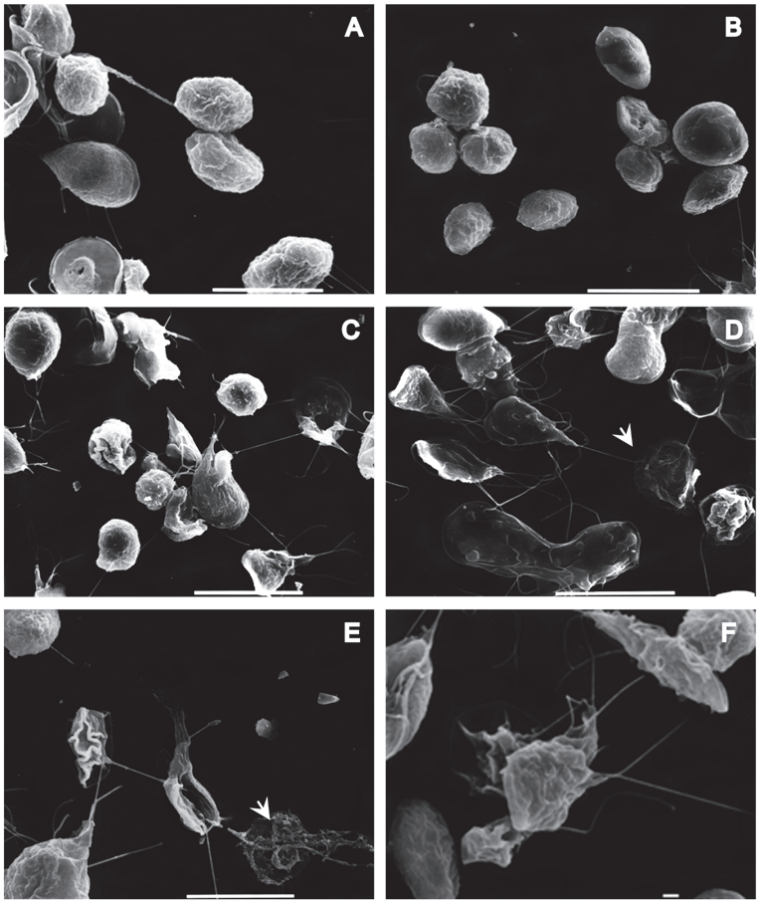
SEM micrographs of *Giardia lamblia* cysts treated with CD, and LA. Samples of cells treated, were processed for SEM as described in methods. 10 µM CD (C, D), 1 µM LA (E, F). DMSO control samples (A, B). Bars are 1 and 10 µm. The arrowheads indicate severe damage in some parasites.

To evaluate the morphological alterations caused by the drugs in trophozoites and cysts, the parasites were processed by SEM. After 72 h of drug-treatment, the trophozoites showed visible morphological changes. Parasites treated with CD appeared rounded, shrunk or inflated with some protrusions in the ventral disk, bearing damaged flagella and caudal region, and the plasma membrane with fractures and wrinkles-like (Figure 6C and D). LA provoked similar effects on *Giardia* as CD did; some trophozoites with irregular form and perforations on the dorsal face were observed (Figure 6E and F). Futhermore, the drugs induced serious alterations on the shape of precysts and cysts. We observed interrupted cytokinesis, precysts with damages in the disc, on the flagella and membrane. The cysts formed unusual aggregates, some of them presented changes at the intermembranal space, also shrank cysts were observed (Figure 7).

In terms of effectiveness, LA was more disastrous for *Giardia* cytoskeleton than CD (Figure 7C and D).; this drug caused a wide variety of abnormal forms, which include parasites without membranes (Figure 7E and F).

## Discussion

This research is a complementary study on *Giardia lamblia* microfilaments, it contributes with new evidence about the role of actin in both processes: growth and encystation. Previous reports, in which heterologous actin antibodies were used, described actin distributed in various specific sites of *Giardia* trophozoites [18], [19]. In this work we used several anti-actin from different sources without any satisfactory result, we used also a monoclonal mouse anti-actin antibody, which has been mostly used and tested to identified actin in plants, mammal cells and other parasites [39], [40], [41], [42], even though we demostrated by Western blotting the specificity of this antibody for actin *Giardia* in trophozoites and cysts, it was not possible by immunolfuorescence to see the actin label in the previously reported structures (data no show). We used also TRITC-phalloidin, largely used for the specific recognition of microfilaments in many species. It has been reported that actin residues E117, M119, G158, R177, D179, M355 and C374 are important for actin-phalloidin interaction [43], [44], [45], [46]. Drouin et al., (1995), found that *Giardia* actin has 58% identity with actin from other species, Woodson and Hawse (2002) by a systematic phylogenetic analysis included actin *Giardia* as a conventional actin. In this study, we confirmed *Giardia* actin identity by performing a BLAST and an alignment with the Vector software, using the sequence for *Giardia* actin (GenBank accession no. P51775) together with actin sequences from *Dictyostelium* (GenBank accession no. EAL62675), Rattus norvegicus ((GenBank accession no. CAA24529), rabbit (GenBank accession no. NP001095153) and human (GenBank accession no. NP001092, NP001605). The result was 99.5% positives and 55.4% identity, also the alignment revealed that *Giardia* actin indeed has the important residues for phalloidin interaction ( Figure S1).

In this study, TRITC-phalloidin revealed actin on the ventral disc, near the nuclei, at the median body, in flagella and like a mesh which occupies the entire trophozoite. Moreover, this paper is the first report about actin distribution changes, accompained by variations in the actin mRNA expression levels in *Giardia* during encystation. Our results suggest that the earlier encystation stages are accompanied by a high dynamic of the actin cytoskeleton and indirectly imply that actin and associated proteins “not necessarily those reported for other systems” could play many different roles in this parasite.

To explore the actin role during *Giardia* growth and encystation, parasites were exposed to drugs that affect the actin dynamics. CD disrupts the actin organization mostly by capping the fast growth end of actin filaments [47]; LA prevents actin assembly by sequestering actin monomers [48] and JAS causes actin disassembly in *in vivo* assays [49]. DMSO was used to solubilize and administrate the drugs. This compound at concentrations higher than 0.2%, can be devastating for intestinal cell lines; however, a 0.2% DMSO concentration does not affect *Giardia* cells [50]. Also, our images revealed no significant changes between non-treated *Giardia* cells related to those treated with 0.1% DMSO.

Some authors reported an effect of CD on *Giardia* growth and morphology [29], even though our experimental conditions were different (the time when the drugs were added and the concentration of DMSO used), our results confirmed a time depending effect of CD, LA and JAS on the growth. DIC and SEM images revealed and confirmed the damage caused by these drugs, meanwhile, the aforementioned work also demonstrate irregular movements in cells treated with CD. In concordance, the results presented in this paper demonstrate that CD and LA provoked severe morphological alterations, among them, the most conspicuous were the shrank caudal region; therefore it suggests that actin participate in the caudal movement.

Our results are the first study about actin in cysts and encystation process. During the encystation process, CD and LA in the medium, caused alterations on the distribution of microfilaments concurrent with morphology alterations and reduction on the ESVs and cysts wall label. Besides, the relative-quantitative RT-PCR analysis demostrated that changes on structured actin by LA indirectly caused a down regulation on CWP1 expression. Other important event was the finding that eventhought actin is considered a constitutive gene in other cells, in the case of *Giardia* this protein was overexpressed during early encystation. All our observations indicate that structured actin have an important role in growth and encystation of *Giardia lamblia*. In summary, in *Giardia lamblia*, actin gives stability to structures, maintains the shape of the parasite, and might be regulating gene expressions as has been described in higher eukaryotes and other parasites [51], [52], [53], also it must have a role in intracellular transport. Obviously, particular ABPs and microtubular associated proteins MAPs must be involved in those processes, and currently our group is addressing that item.

## Supporting Information

Figure S1 Alignment of Giardia actin against other-species actins. Alignment of Giardia actin against other-species actins. The boxes depict the residues involved in actin-phalloidin interaction identificated on actin Giardia sequence.
(5.37 MB TIF)
