# Supplementary figures and images for: Participation of Actin on Giardia lamblia Growth and Encystation
Source: PLoS One. 2009 Sep 23;4(9):e7156. doi: 10.1371/journal.pone.0007156 (PMC2743995; doi:10.1371/journal.pone.0007156)

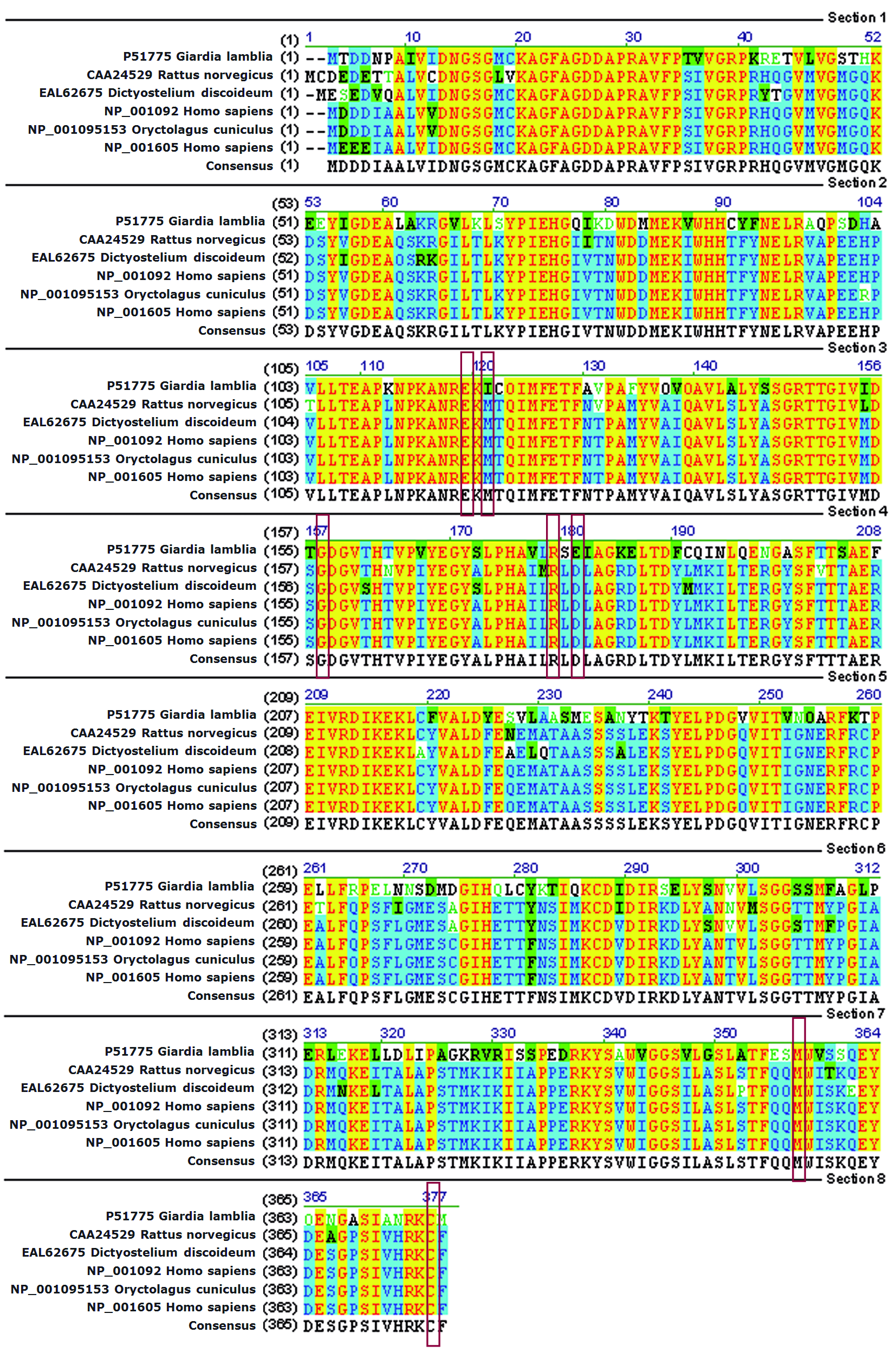

Supplement: Figure S1 — Alignment of Giardia actin against other-species actins. Alignment of Giardia actin against other-species actins. The boxes depict the residues involved in actin-phalloidin interaction identificated on actin Giardia sequence. (5.37 MB TIF) [file pone.0007156.s001.tif]
